# Genomic Prediction of Grain Yield in a Barley MAGIC Population Modeling Genotype per Environment Interaction

**DOI:** 10.3389/fpls.2021.664148

**Published:** 2021-05-24

**Authors:** Damiano Puglisi, Stefano Delbono, Andrea Visioni, Hakan Ozkan, İbrahim Kara, Ana M. Casas, Ernesto Igartua, Giampiero Valè, Angela Roberta Lo Piero, Luigi Cattivelli, Alessandro Tondelli, Agostino Fricano

**Affiliations:** ^1^Dipartimento di Agricoltura, Alimentazione e Ambiente (Di3A), Università di Catania, Catania, Italy; ^2^Council for Agricultural Research and Economics–Research Centre for Genomics and Bioinformatics, Fiorenzuola d’Arda, Italy; ^3^Biodiversity and Crop Improvement Program, International Center for Agricultural Research in the Dry Areas, Avenue Hafiane Cherkaoui, Rabat, Morocco; ^4^Department of Field Crops, Faculty of Agriculture, University of Cukurova, Adana, Turkey; ^5^Bahri Dagdas International Agricultural Research Institute, Konya, Turkey; ^6^Aula Dei Experimental Station (EEAD-CSIC), Spanish Research Council, Zaragoza, Spain; ^7^DiSIT, Dipartimento di Scienze e Innovazione Tecnologica, Università del Piemonte Orientale, Vercelli, Italy

**Keywords:** genomic prediction, MAGIC, barley, GBLUP, genotype x environment interaction

## Abstract

Multi-parent Advanced Generation Inter-crosses (MAGIC) lines have mosaic genomes that are generated shuffling the genetic material of the founder parents following pre-defined crossing schemes. In cereal crops, these experimental populations have been extensively used to investigate the genetic bases of several traits and dissect the genetic bases of epistasis. In plants, genomic prediction models are usually fitted using either diverse panels of mostly unrelated accessions or individuals of biparental families and several empirical analyses have been conducted to evaluate the predictive ability of models fitted to these populations using different traits. In this paper, we constructed, genotyped and evaluated a barley MAGIC population of 352 individuals developed with a diverse set of eight founder parents showing contrasting phenotypes for grain yield. We combined phenotypic and genotypic information of this MAGIC population to fit several genomic prediction models which were cross-validated to conduct empirical analyses aimed at examining the predictive ability of these models varying the sizes of training populations. Moreover, several methods to optimize the composition of the training population were also applied to this MAGIC population and cross-validated to estimate the resulting predictive ability. Finally, extensive phenotypic data generated in field trials organized across an ample range of water regimes and climatic conditions in the Mediterranean were used to fit and cross-validate multi-environment genomic prediction models including G×E interaction, using both genomic best linear unbiased prediction and reproducing kernel Hilbert space along with a non-linear Gaussian Kernel. Overall, our empirical analyses showed that genomic prediction models trained with a limited number of MAGIC lines can be used to predict grain yield with values of predictive ability that vary from 0.25 to 0.60 and that beyond QTL mapping and analysis of epistatic effects, MAGIC population might be used to successfully fit genomic prediction models. We concluded that for grain yield, the single-environment genomic prediction models examined in this study are equivalent in terms of predictive ability while, in general, multi-environment models that explicitly split marker effects in main and environmental-specific effects outperform simpler multi-environment models.

## Introduction

The experimental design that underlies Multi-parent Advanced Generation Intercrosses (MAGIC) populations traces its origins to the advanced inter-cross lines, which were originally developed in animal model species (Yalcin et al., 2005). MAGIC populations are developed crossing multiple inbred parents or founders, which are subsequently inter-mated several times following pre-defined crossing schemes to shuffle founder genomes in each single line (Huang et al., 2015). In plants, MAGIC populations have been explicitly developed for genetic research purposes as they allow to increase power and precision for detecting and mapping quantitative trait loci (QTLs) (Cavanagh et al., 2008; Huang et al., 2015; Scott et al., 2020). Theoretically, MAGIC populations have the potential to dissect the genetic bases of complex traits at sub-centimorgan scale, allowing to overcome common issues related to the use of biparental families for QTL mapping and detection such as low-resolution power, low genetic diversity of parents and limited number of recombination events (Valdar et al., 2006). In cereal crops, MAGIC populations have been developed and established for rice (Bandillo et al., 2013; Ponce et al., 2018), bread wheat (Mackay et al., 2014; Sannemann et al., 2018; Stadlmeier et al., 2018), maize (Dell’Acqua et al., 2015; Jiménez-Galindo et al., 2019) and barley (Mathew et al., 2018) and to date they have been deployed for unraveling the genetic bases of biotic and abiotic stresses, grain yield (GY) and seed quality traits. Beyond the aforementioned applications, barley MAGIC populations have been recently exploited to disentangle the effect of epistasis on flowering time (Mathew et al., 2018; Sannemann et al., 2018; Afsharyan et al., 2020).

Similarly to MAGIC, the theory underlying genomic prediction (GP) was originally developed and deployed in animal species. The pivotal component of GP is a population of individuals having phenotypic and genotypic information, which is known as training population (TP) and is used to regress genome-wide single nucleotide polymorphisms (SNPs) or other types of DNA markers on phenotypes to simultaneously predict their effects (Meuwissen et al., 2001), that is for training GP models. Trained GP models are subsequently used in combination with the genotypic information of candidate individuals that must be selected for computing their genomic estimated breeding values (GEBVs) and ranking them to apply truncation selection (Meuwissen et al., 2001; Heffner et al., 2009). This latter population of candidate individuals having only genotypic information is known as breeding population (BP) (Meuwissen et al., 2001; Heffner et al., 2009). To date, GP has been largely applied for crop improvement fitting GP models trained with individuals from either biparental families or diversity panels of mostly unrelated accessions. As the genetic relatedness of TP and BP affects the prediction ability of GP models (Ben Hassen et al., 2018; Norman et al., 2018), these two approaches have profound differences in terms of versatility as DNA marker effects estimated on diversity panels have the potential of a broader applicability and might be used in different breeding programs (Bassi et al., 2015), while GP models trained with individuals of biparental families can allow to accurately predict the performance of offspring produced within the same cross.

Typically, GP models require to regress a number of predictors (DNA markers) that greatly exceeds the number of observations or phenotypes and several parametric and non-parametric models have been proposed to deal with overfitting and the “large *p*, small *n*” problem (Meuwissen et al., 2001; Jannink et al., 2010; Pérez and de los Campos, 2014) as in these conditions the estimation of marker effects using ordinary least squares method is not practicable. A commonly used solution is to estimate marker effects jointly using the Least Absolute Shrinkage and Selection Operator (LASSO) method (Tishbirani, 1996) and its Bayesian counterpart (Bayesian Lasso or BL), which uses a penalizing or regularization parameter (λ) that denotes the amount of shrinkage for regressing markers (De Los Campos et al., 2009). Other popular whole genome regression methods based on Bayesian theory are BayesA and BayesB (Meuwissen et al., 2001), which relax the assumption of common variance across marker effects adopted in other models (e.g., ridge regression) and allow each marker to have its own variance. Differently to BayesA, BayesB allows having markers with no effects in the model and theoretically assumes more realistic conditions as it is plausible that a large fraction of genome-wide markers does not contribute to explaining the observed phenotypic variance. Beyond these methods, whole genome regression based on reproducing kernel Hilbert space (RKHS) has been proposed and applied to implement GP models (Gianola and Van Kaam, 2008; Gota and Gianola, 2014). In the RKHS regression, a reproducing kernel, that is any positive definite function for mapping from pairs of points in input space to other pairs of points, is used to transform DNA markers of individuals in square distance matrix that are used in a linear model (Gota and Gianola, 2014). The Gaussian Kernel (GK) is one of the most common function used as reproducing kernel and depends on the bandwidth (or smoothing) parameter *h* that controls the decay rate of the kernel as two points step away. Several studies have shown that the use of GK in combination with RKHS improves the prediction of genetic values if the bandwidth parameter *h* is correctly chosen (Pérez-Elizalde et al., 2015). Moreover as RKHS regression does not assume linearity, this model might allow to better capture non-additive effects without explicitly including epistatic interactions and dominance in GP models (Gianola and Van Kaam, 2008). Differently from methods based on whole genome regression of markers, the genomic best linear unbiased prediction (GBLUP) method treats genomic values of individuals as random effects in a linear mixed model and uses a genomic relationship matrix based on DNA marker data to compute GEBVs (VanRaden, 2008; Wang et al., 2018). Notably, the use of RKHS along with the genomic relationship matrix is equivalent to the mixed linear model of GBLUP, that is GBLUP method represents a special case of RKHS regression (Gota and Gianola, 2014).

The effectiveness of GP depends, among other factors, on the degree of correlation between GEBVs and true genetic values that is the predictive ability of the model. In practice, the predictive ability is evaluated using the Pearson’s correlation coefficient between GEBVs and the realized phenotypes or other estimators (e.g., adjusted means). To date several empirical studies have been conducted for fitting GP models on biparental populations and panels of mostly unrelated accessions across different species and traits, which point out that, depending on the genetic architecture of the trait, each statistical model has its own advantages and disadvantages in term of predictive ability and estimation of marker effects (Heslot et al., 2012; Ben Hassen et al., 2018). Other factors that strongly influence the predictive ability are the size of the TP, its structure, and its relatedness with the BP (Desta and Ortiz, 2014). Several targeted and untargeted methods have been developed to optimize the composition of TP for maximizing the predictive ability for a given set of individuals (Rincent et al., 2012; Akdemir et al., 2015). Nevertheless, these methods generally generate trait-dependent TPs which might hamper the implementation of these procedures in real breeding programs.

The first objective of the present study was to create a new barley MAGIC population using a diverse founder set of old and new 6-rowed, winter cultivars showing contrasting GY, which was examined across an ample range of site-by-season combinations characterized by different temperature and precipitation patterns. The second objective of this study was to combine data collected across these field trials with genotypic information to fit different single-environment genomic prediction (SE-GP) and multi environment genomic prediction (ME-GP) models for empirically assessing the predictive ability in multi-parent populations. Moreover, we applied different untargeted optimization methods to this MAGIC population for assembling and benchmarking the performance of optimized TPs. Fitting SE-GP and ME-GP models to MAGIC lines, we aimed at broadening the use of these experimental populations beyond classical QTL mapping and analysis of epistatic effects for sustaining and accelerating barley breeding.

## Materials and Methods

### Development of the Barley MAGIC Population

The MAGIC population used in this study was developed using a founder set of eight 6-rowed barley genotypes with a winter growth habit, which were selected on the basis of their pedigrees and similarity in days-to-heading (DH) (Table 1). At the first stage of MAGIC development, four F_1_ populations were created crossing one of the four old 6-rowed barley varieties (Hatif de Grignon, Dea, Robur and Athene) with one of the four 6-rowed modern barley varieties (Ponente, Ketos, Aldebaran and Fridericus). At the second stage of MAGIC development, half-diallel crosses of these four F_1_ individuals were carried out to generate six sets of plants. Finally, these six sets of genotypes, each of which contained the alleles of four out eight founder parents, were appropriately crossed in predefined funnel schemes to combine the genome of the eight founders in single lines. Differently from the original crossing schemes developed for constructing MAGIC populations (Cavanagh et al., 2008), instead of recursively self-fertilizing these plants for several generations, seeds of the eight-way inter-crosses were sent to an external lab (SAATEN-UNION GmbH, Germany) to generate 352 inbred MAGIC lines using doubled haploid technology.

**TABLE 1:** Founder set of barley varieties that were intermated for creating the barley MAGIC population.

Genotype	Year of release	Country of release	Pedigree	DH (days)	PH (cm)	GY (t/ha)
Hatif de Grignon	1937	France	Selection from French landraces	208.3	95.9	4.1
Dea	1953	Germany	[(Ragusa x Peragis12) × (Heils Franken × Frw.Berg)] x [(Ragusa × Mahnd.Viktoria) (Ragusa × Bolivia)]	212.1	95.3	6.0
Robur	1973	France	Ager × (Hatif de Grignon × Ares)	208.3	78.8	6.3
Athene	1977	Germany	(Herfodia × Hord.sp.nigrum H204) × (Madru x Weissenhaus-Stamm)	211.5	94.0	6.0
Ponente	2001	Italy	(Vetulkio × Arma) × Express	209.7	85.0	6.3
Ketos	2002	France	(Gotic x Orblonde) × (12813 × 91H595)	208.6	81.9	6.8
Aldebaran	2003	Italy	Rebelle × Jaidor	208.5	83.0	7.2
Fridericus	2006	Germany	Carola × LP 6–564	211.6	89.3	7.3

*For each genotype of the founder set, the adjusted means of days to heading (DH), plant height (PH) and grain yield (GY) scored in eight different trials were reported along with available pedigree information.*

### Field Trials and Plant Phenotyping

The MAGIC population of 352 inbred individuals and the eight founder parents (Table 1) were sown during the fall of two consecutive growing seasons (2015–2016 and 2016–2017) in Fiorenzuola d’Arda (Italy) at CREA-Centro di Genomica e Bioinformatica (44°55′39.0”N 9°53′40.6”E, 78 m above sea level), using an alpha-lattice design with two-replicates. The whole set of MAGIC and the founder parents were also sown during the fall of 2015–2016 growing season in Marchouch (Morocco) at the Experimental station (33°36′43.5” N 6°42′53.0”W, 390 m above sea level) of the “International Center for Agricultural Research in the Dry Areas” using the same experimental design. Similarly, the subset of 82 MAGIC lines included in the optimized TP (TP-Diverse) and the eight founder parents were sown during the fall in 2017–2018 and 2018–2019 growing seasons in Fiorenzuola d’Arda under two different levels of nitrogen fertilization using alpha lattice experimental designs with two replicates. Trials conducted under ideal nitrogen conditions were fertilized with 100 kg/ha of nitrogen applied in two doses: 50 kg/ha were used at the sowing and 50 kg/ha were applied at the stem elongation stage. Field trials conducted under low nitrogen conditions received 50 kg/ha of nitrogen, 25 of which were applied at sowing while the remaining amount was applied at the stem elongation stage. In the growing season 2018–2019, other two field trials were conducted in Konya (Turkey) (37°53′37.9”N 32°37′26.0”E, 1,005 m above sea level) and in Adana (Turkey) (36°59′52.9”N 35°20′28.0”E, 24 m above sea level) to phenotype the optimized TP (TP-Diverse) using the same experimental design. For each trial considered in this study, plots of three square meters and a sowing density of 350 seeds per square meter were adopted, respectively. Local check cultivars were included as internal checks in all experiments to compare phenotypes with trait observations collected in past seasons. Common protocols were adopted for each trial to phenotype plant genotypes for GY and DH. Phenotyping of MAGIC lines for GY was conducted as follows: from each plot grains were collected using a combine harvester and the total grain weight recorded in each plot was converted in tons per hectare. DH was measured as the number of days between sowing date and the date of heading stage, which was defined when 50% of the plants in a plot were at Zadoks’ 55 growth stage (Zadoks et al., 1974). For each trial, phenotypic data of GY used in GP models were centered by subtracting the overall mean and standardized dividing by the sample standard deviation.

### Statistical Models for Computing the Adjusted Means of GY

The adjusted means of GY were computed in each site-by-season combination and across environments including DH as fixed covariate using the approach described in Emrich et al., 2008. The resulting model for computing the adjusted means of GY collected in field trials organized according to alpha-lattice design was:

(1) $$y_{\mathrm{ijk}}=1\mu +{\mathrm{Rep}}_{i}+{\mathrm{Block}}_{j}\left({\mathrm{Rep}}_{i}\right)+{\mathrm{Gen}}_{k}+{\mathrm{DH}}_{k}+e_{\mathrm{ijk}}$$

where y_ijk_ is the response variable, that is the raw GY, μ is the general mean, Rep_i_ is the effect of the *i*^*th*^ replicate, Block_*j*_(Rep_*i*_) is the effect of the^*jth*^ incomplete block within the *i*^*th*^ replicate, Gen_*k*_ is the random effect of the *k*^*th*^ genotype and DH is the effect of “Days-to-heading” covariate measured in each plot. In this model it is supposed that the random effects of Gen_*k*_ follow a normal distribution with mean 0 and variance $\sigma_{g}^{2}$, that is ${\text{Gen}}_{k}∼NIID\left(0,\sigma_{g}^{2}\right)$, and similarly, the residual terms e_ijk_ are normally distributed with mean 0 and variance equals to σ^2^, that is e_ijk_∼*NIID*(0,σ^2^). The adjusted GY values obtained predicting the random terms Gen_*k*_ from the aforementioned model were used as phenotypes for training GP models. The linear mixed model reported in Equation 1 was fitted for each site-by-season combination using R 3.6.2 statistical environment and lme4 package (Bates et al., 2015) and variance components of fitted models were used to compute broad sense heritability (H^2^) of GY.

### Genotyping of Genetic Materials

DNA was extracted from plant leaves using the Macherey Nagel Plant II extraction kit (Macherey Nagel, Dueren, Germany) and analyzed using gel electrophoresis and Quant-iT^TM^ PicoGreen^TM^ dsDNA Assay Kit (ThermoFisher, Grand Island, NY, United States) following manufacturer’s instructions to assess quality and concentration, respectively. DNA samples were shipped to a propel-certified service provider (Trait Genetics GmbH, Gatersleben, Germany) and fingerprinted using the Illumina Infinium technology along with the Barley 50 k iSelect SNP Array (Bayer et al., 2017). To update the physical positions of SNP markers interrogated with the Barley 50 k iSelect SNP Array, probe sets used to design this array were mapped against the new reference sequence of barley (Monat et al., 2019). The raw genotyping table was imported in R software using “synbreed” package (Wimmer et al., 2012) to filter out markers with more than 10% of missing data and impute remaining missing data using Beagle 4.1 (Browning and Browning, 2016). 20 random leaf samples from field trials organized in Adana and Marchouch were genotyped using Illumina Infinium technology and Barley 50 k iSelect SNP Array to assess whether mislabelling of genotypes occurred during phenotyping operations and data collection.

### Clustering and Linkage Disequilibrium Analyses of the MAGIC Population

Principal component analysis was used to assess the diversity of the whole MAGIC population and was carried on imputed SNP data of the 352 MAGIC lines and the eight founders using ade4 package along with R version 3.6.2 (Thioulouse et al., 2018; R Core Team, 2019)., 2018). The first two principal components were used to visualize the dispersion of MAGIC lines in a graph. Linkage disequilibrium between pairs of markers was measured using *r*^2^ (Hill and Robertson, 2008) in the subset of MAGIC genotypes included in the optimized TP and computed using Plink 1.9 software (Purcell et al., 2007; Chang et al., 2015).*r*^2^ values showing *p*-values above 0.001 were filtered out, while the remaining pairwise *r*^2^ values were imported and examined with a custom script developed for R 3.6.2 (R Core Team, 2019) to compute the mean *r*^2^ in 100 kb windows, which was plotted in R 3.6.2 using ggplot2 package (Wickham, 2016).

### Statistical Models Used for Fitting SE-GP

SE-GP models were fitted using BayesA, BayesB and BL models (Tishbirani, 1996; Meuwissen et al., 2001; Park and Casella, 2008). Moreover, RKHS regression models were fitted using a linear GBLUP kernel (GB) and a non-linear GK (Gianola and Van Kaam, 2008; Gota and Gianola, 2014). For the GK, that is K(xi,xi′)=e-(h*dii2)′, where $d_{ii}^{2}{}^{\prime }$ points out the squared Euclidean distance between individuals *i* and *i*′, the rate of decay imposed by the bandwidth parameter *h*, was estimated using an empirical Bayesian methodology (Pérez-Elizalde et al., 2015) modifying published R codes (Cuevas et al., 2016).

### Statistical Models Used for Fitting ME-GP

Beyond SE-GP models, the adjusted means of GY computed across different site-by-season combinations were fitted to three previously described ME-GP models. Following the model nomenclature reported in Bandeira e Sousa et al. (2017), these three models were indicated in this study as “multi-environment, main genotypic effect” (MM) model (Jarquín et al., 2014; López-Cruz et al., 2015; Bandeira e Sousa et al., 2017), “multi-environment, single variance G×E deviation model” (MDs) (Jarquín et al., 2014; Bandeira e Sousa et al., 2017) and the “multi-environment, environment-specific variance G×E deviation model” (MDe) (López-Cruz et al., 2015; Bandeira e Sousa et al., 2017). Site-by-season combinations were considered as environments in MM, MDs and MDe regression models, which are briefly defined and summarized as follows. In the MM model, environments were considered as fixed effects while the random genetic effects were considered constant across all environments without modeling marker x environment interactions. Following matrix notation, the MM regression model is defined as follows:

(2) $$y=1\mu +Z_{e}\beta_{e}+Z_{u}u+\varepsilon$$

where y is the vector of observations collected in all environments, is the overall mean, Z_*e*_ is the incidence matrix that connects observed phenotypes to the environments in which they were measured, β_*e*_ is the vector of environmental fixed effects that must be estimated, Z_*u*_ is an incidence matrix connecting genotypes with phenotypes for each environment, u is the vector of random genetic effects that must be predicted while ε is a vector of model residuals. In this model, marker genetic effects are assumed as $\text{u}∼N\left(0,\sigma_{\mu 0}^{2}K\right)$, that is, they follow a multivariate normal distribution with mean and variance-covariance matrix equal to zero and $\sigma_{\mu 0}^{2}K$, respectively. The term $\sigma_{\mu 0}^{2}$ of the variance-covariance matrix is the variance of additive genetic effects across environments, while *K* can be either a genomic relationship matrix (VanRaden, 2008) or a kernel function as discussed below. Model residuals of the vector are assumed to be independent and normally distributed with null mean and variance equal to$\sigma_{e}^{2}$, that is$\varepsilon ∼N\left(0,I\sigma_{e}^{2}\right)$, where *I* points out the identity matrix. Overall, the MM regression model estimates marker effects across all environments and does not split them in main marker effects and in environmental-specific effects as in MDs and MDe models. As already substantiated in López-Cruz et al. (2015), for balanced field trial designs, MM is equivalent to fitting a genomic regression model using the average performance of each line across environments as phenotype.

Differently from the MM model, the MDe model allows markers to assume different effects in each *j*^*th*^ environment (López-Cruz et al., 2015; Bandeira e Sousa et al., 2017), and consequently allows to account for marker x environment interactions. This model assumes that the effects of the *j*^*th*^ environments, and the effects of markers are separated into two components, which are the main effect of markers for all environments, names as *b*_*0k*_, and the peculiar random effect *b*_*ik*_, of the markers in each *j*^*th*^ environment, that is the effects of marker x environment interactions (López-Cruz et al., 2015). Consequently, in MDe models, the effect of the *k*^*th*^ marker on the *j*^*th*^ environment (β_*jk*_) is described as the sum of an effect common to all environments (*b*_*0k*_), plus a random deviation (*b*_*ik*_) peculiar to the *j*^*th*^ environment, that is β_*jk*_ = *b*_0*k*_ + *b*_*ik*_.

Following matrix notation, the MDe regression model is defined as follows:

(3) $$y=1\mu +Z_{e}\beta_{e}+Z_{u}u_{o}+u_{E}+\varepsilon$$

where, Z_*e, e*_ have the same meaning of the MM regression model, u_*o*_ represents the main effect of markers across all environments with a variance–covariance structure similar to MM model, that is, $\text{u}N\left(0,\sigma_{\mu 0}^{2}K\right)$. As pointed out by López-Cruz et al. (2015)$\sigma_{\mu 0}^{2}$ is common to all environments, and the borrowing of information among environments is generated through the kernel matrix *K*. u_*E*_ points out the specific effects of marker x environment interactions, which follow a multi-variate normal distribution with null mean and a variance–covariance matrix*K*_*E*_, that is, u_*E*_*N*(0,*K*_*E*_). For *j* environments, the variance-covariance matrix *K*_*E*_ is defined as follows:

$$K_{e}=\left[\begin{array}{ccccc} \sigma_{\mu E1}^{2}K_{1} & \cdots & 0 & \cdots & 0\\ \vdots & \ddots & \vdots & \ddots & \vdots \\ 0 & \cdots & \sigma_{\mu Em}^{2}K_{m} & \cdots & 0\\ \vdots & \ddots & \vdots & \ddots & \vdots \\ 0 & \cdots & 0 & \cdots & \sigma_{\mu Ej}^{2}K_{j} \end{array}\right]$$

As explained in Bandeira e Sousa et al. (2017), *K_E_* can be discomposed as a sum of *j* matrices, one for each *j* environment. Consequently, the interaction term u_*E*_ can be decomposed in *j* environmental specific effects to transform equation 3 as follows:

(4) $$y=1\mu +Z_{e}\beta_{e}+Z_{u}u_{0}+u_{E1}+u_{E2}+u_{E3}+\ldots +u_{Ej}+\varepsilon$$

where each interaction effect u_*Ej*_ has a normal distribution with null mean and a variance-covariance structure $\sigma_{\mu Ej}^{2}K_{j}$.

Starting from the MM regression model, the MDs model adds the random interaction effect of the environments with the genetic information of the lines pointed out with ue. Following matrix notation, the MDs modes is described as follows:

(5) $$y=1\mu +Z_{e}\beta_{e}+Z_{u}u+ue+\varepsilon$$

where, Z_*e*_, β_*e*_, Z_*u*_, u and ε have the same meaning of the MM regression model. As substantiated in Jarquín et al. (2014) the interaction term ue has a multi-variate normal distribution with null mean and variance-covariance matrix equal to ${\left[Z_{u}KZ_{u}^{\prime }\right]}^{{}^{\circ}}\left[Z_{E}Z_{e}^{\prime }\right]$, where the Haddamar product operator denotes the element to element product between the two matrices in the same order.

In the present study, MM, MDs and MDe regression models were fitted using either the linear GB kernel method (VanRaden, 2008) or the non-linear GK method (Bandeira e Sousa et al., 2017). For the linear GB kernel method, the matrix *K* of the aforementioned models was the genomic relationship matrix and was computed as $K=\left(\frac{XX^{\prime }}{p}\right)$ (VanRaden, 2008), where X is the standardized matrix of molecular markers for the individuals, of order *n* by *p*; where *n* and *p* are the number of observations and the number of markers, respectively. For GK method, the matrix *K* of MM, MDs and MDe regression models was computed as Kj(xij,xij′)=e-(hj*dii2)′ where $d_{ii}^{2}{}^{\prime }$ is the squared Euclidean distance of the markers genotypes in individuals *i* and *i*′’ for the *j*^*th*^ environment. Similarly to SE-GP models, the bandwidth parameter *h* was computed using an empirical Bayes method (Pérez-Elizalde et al., 2015; Cuevas et al., 2016).

MM, MDs and MDe regression models used in this study were fitted using BGLR package 1.08 (Pérez and de los Campos, 2014) in R 3.6.2 statistical environment, adapting scripts provided in the framework of other studies (Bandeira e Sousa et al., 2017). For each model implemented in this study, predictions were based on 500,000 iterations collected after discarding 10,000 iterations for burn-in period-and using a thinning interval of five iterations. Trace plots for each of the variance parameters were created to assess whether the number of burn-in iterations was sufficient.

### Optimization of the TPs

In this study three different untargeted optimization criteria based on coefficient of determination (Laloe, 1993), predictive error variance (Rincent et al., 2012) and rScore (Ou and Liao, 2019) were used to assemble three corresponding TPs, each of which groups a set of 90 MAGIC individuals. The R package TSDFGS (Ou and Liao, 2019) was used to assemble these three optimized TPs using the aforementioned criteria. A fourth empirical untargeted optimization criterion was adopted for assembling another TP from the whole MAGIC population and aimed at maximizing the average distance between each selected accession and the closest other line using the modified Roger’s distance (Thachuk et al., 2009). This criterion was implemented in R 3.6.2 using the heuristic algorithm implemented in the package Core Hunter3 (De Beukelaer et al., 2018) and was used to select a subset of 82 out 352 MAGIC individuals along with the eight MAGIC founder parents.

### Cross Validation Schemes

In this study several cross-validation (CV) schemes were adopted for estimating the predictive ability of GP models along with their standard errors (Burgueño et al., 2012; Gianola and Schon, 2016). For estimating the predictive ability of SE-GP models implemented with BayesA, BayesB, Bayesian Lasso, GB and RKHS with GK, cross validation was carried out using 100 repeated random partitioning of MAGIC population into training and validation sets. Using increasingly larger TPs of 80, 90, 100, 110, 120, 130, 140, 150, and 160 individuals, CV schemes were applied to compute mean and standard deviation of predictive ability for each TP size. Totally 4,500 models were fitted to carry out this CV experiment, combining the five statistical models with the aforementioned dimensions of the TP and 100 repeated random partitioning of MAGIC in training and validation sets.

Cross-validation of SE-GP models fitted using optimized TPs was carried out using the standard leave-one-out (LOO) strategy to estimate their predictive ability (Gianola and Schon, 2016). Basically, using LOO strategy, N GP models are fitted using N-1 individuals excluding recursively one individual from the TP and the GEBV of the excluded line is predicted from a model trained using all other lines. In our LOO experiment, this was carried out separately for each group of 90 lines included in the optimized TPs, and the accuracy of these predictions was calculated as the Pearson’s correlation coefficient between GEBVs and the corresponding adjusted means of GY.

The predictive ability of ME-GP models was assessed using cross-validation 1 (CV1) and cross-validation 2 (CV2) schemes (Burgueño et al., 2012), assigning 90% of lines to the training set and the remaining 10% to the validation set. In both CV schemes, all the parameters of the MM, MDs and MDe regression models were recursively re-estimated in each of 100 random partitions. For each random partitioning, models were fitted using genotypes included in the training sets and the predictive ability was computed as the Pearson’s correlation coefficient between GEBVs and the corresponding adjusted means of GY. Overall, 100 Pearson’s correlations were computed for each model and the mean and standard deviation of these values were computed to estimate the predictive ability of GP models.

## Results

### Development of the Barley MAGIC Population

The barley genotypes included in the founder set of MAGIC were examined in field trials organized in height site-by-season combinations in Italy, Germany and Scotland (Xu et al., 2018) for assessing the diversity of European cultivars for GY, plant height and DH. These field trials showed that the founder set, which includes four elite and four old barley varieties with different genetic background, exhibits limited variation of DH values (Table 1). Following a modified version of the standard crossing design (Huang et al., 2015), this founder set was intermated to create an eight-way MAGIC population of 352 individuals, which were subsequently genotyped to assess the contribution of each founder parent to the mosaic genome of each line.

### Estimating the Predictive Ability of GP Models as a Function of TP Size

In GP models, the variation of predictive ability as a function of the TP size has been empirically investigated on segregating families and in collections of mostly unrelated accessions (Norman et al., 2018). Here, we investigated the relationship between TP size and the predictive ability of different GP statistical models fitted to the barley MAGIC population. To carry out this analysis, the whole panel of 352 MAGIC lines and the founder parents were genotyped using the Barley 50 k iSelect SNP Array (Bayer et al., 2017). SNPs with more than 10% of missing data were discarded, while the remaining missing genotypes were imputed using the algorithm implemented in BEAGLE (Browning and Browning, 2016). This procedure allowed to identify 19,723 polymorphic SNPs, which were combined to the adjusted means (BLUPs) of GY computed in three site-by-season combinations (Table 2) to fit and cross-validate SE-GP models. Overall, five different whole genome regression methods based on BayesA, BayesB, BL, GB and RKHS fitted with the non-linear GK (Gianola and Van Kaam, 2008; Gota and Gianola, 2014; Cuevas et al., 2016; Crossa et al., 2017) were compared.

**TABLE 2:** Field trials carried out for phenotyping the whole MAGIC population and the founder set for GY.

Acronym	Site	Country	Growing season	Populations	Traits
Fio16IN	Fiorenzuola d’Arda	Italy	2015–2016	352 MAGIC and the founder set	DH, GY
Fio17IN	Fiorenzuola d’Arda	Italy	2016–2017	352 MAGIC and the founder set	DH, GY
Mar16IN	Marchouch	Morocco	2015–2016	352 MAGIC and founder set	DH, GY

**FIGURE 1:**
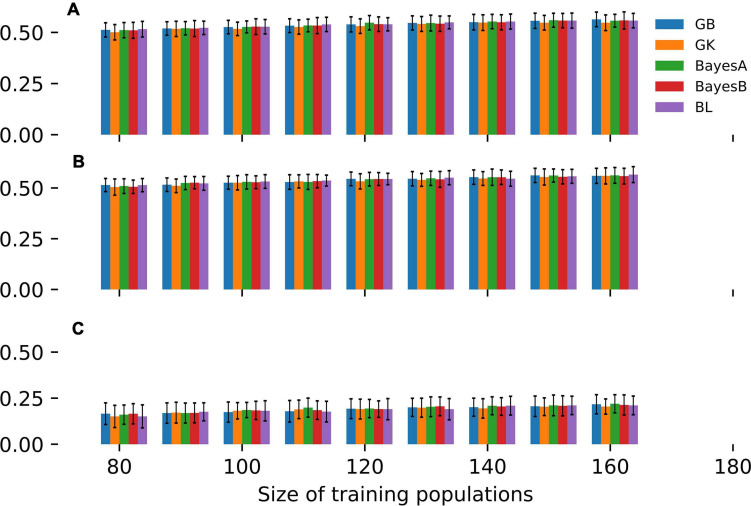
CV of different SE-GP models fitted to GY measured in the MAGIC population. Bars report the values of predictive ability for GY computed in **(A)** Fio16IN, **(B)** Fio17IN, and **(C)** Mar16IN. Bars of different colors point out values of predictive ability computed using GB, GK, BayesA, BayesB and BL models as a function of TP sizes, while the error bars point out the standard deviation of predictive ability values.

These aforementioned SE-GP models were fitted to the MAGIC population and cross-validated for estimating the trend of predictive ability as a function of TP size (Figure 1). Specifically, CV was implemented randomly partitioning 100 times the whole panel of MAGIC lines in a TP and in a validating population (VP). Overall, nine different CV experiments were carried out, using TP sizes of 80, 90, 100, 110, 120, 130, 140, 150, and 160 MAGIC lines and the remaining genotypes as VPs (Figure 1). The CV of these GP models points out that in the three site-by-season combinations (Table 2), GB, GK, BayesA, BayesB and BL show comparable predictive abilities across the entire range of TP sizes considered (Figure 1). Moreover, these CV experiments point out that in temperate locations (Fio16IN, Fio17IN, Table 2), the predictive ability of SE-GP models exceeds 0.50 even using TPs of 80 or 90 individuals (Figure 1), while in the harsh and pre-desertic environment of Mar16IN (Table 2), it does not exceed 0.25 and shows larger standard deviation. Varying the size of TPs from 80 to 160 individuals slightly increases the values of predictive ability for GY in the remaining individuals of the MAGIC population (Figure 1 and Supplementary Table 1) as already substantiated in other GP models fitted using collection of mostly unrelated genotypes (Norman et al., 2018). Overall, this empirical analysis shows that 80 or 90 MAGIC individuals are sufficient to fit SE-GP models yielding high values of predictive ability and that larger TPs do not significantly improve the predictive ability of GP models either in temperate or stressful environments (Figure 1 and Supplementary Table 1).

### Designing Optimized TPs of MAGIC

The predictive ability of GP models fitted in collection of mostly unrelated accessions and in biparental populations depends on the size of TP, the genome distribution and number of molecular markers used for whole genome regression, the genetic composition of TP and its genetic relationship with the BP (Heffner et al., 2009; Jannink et al., 2010; Desta and Ortiz, 2014; Berro et al., 2019). Particularly, it was assessed that using a large reference panel of accessions, the predictive ability of GP models can be improved increasing the diversity of the TPs (Norman et al., 2018). Along with these empirical findings, several statistical criteria and algorithms have been proposed to optimize TPs for maximizing predictive ability using reference panels of accessions or sets of advanced lines (Akdemir et al., 2015; Berro et al., 2019; Ou and Liao, 2019).

**FIGURE 2:**
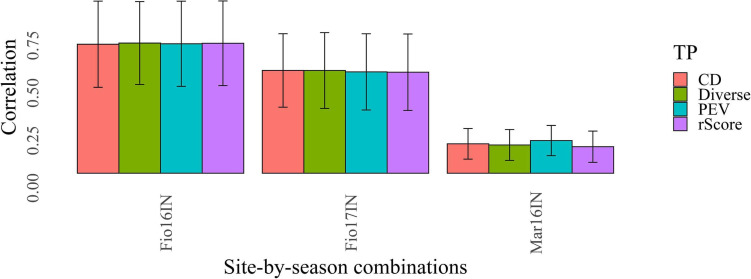
Benchmarking of different methods for optimizing TPs of MAGIC. Bars of different colors report the values of predictive ability obtained with GP models fitted using CD_mean (CD), prediction error variance (PEV), rScore and Diverse optimization criteria. The error bars of each plot point out the standard deviation of the predictive ability values.

Here, we examined three different untargeted optimization criteria based on the coefficient of determination (CD_mean) (Laloe, 1993), prediction error variance (PEV) (Rincent et al., 2012) and rScore (Ou and Liao, 2019) and benchmarked them against a method that samples a diverse TP from the whole MAGIC population using SNP markers (Figure 2). The rationale of this latter method is to maximize the average distance, computed using the modified Roger’s method, between each selected accession and the closest other genotype (Thachuk et al., 2009). This criterion, named entry-to-nearest entry was maximized with a heuristic algorithm to construct a highly diverse TP in which all MAGIC lines are maximally different (De Beukelaer et al., 2018). The TP assembled with this latter untargeted optimization criterion, named “TP-Diverse” (Figure 2), was constructed using the panel of 19,723 polymorphic SNPs detected in the whole MAGIC population, and was subsequently used as optimized TP and benchmarked to TPs assembled using CD_mean, PEV and rScore optimization methods (Figure 2).

**FIGURE 3:**
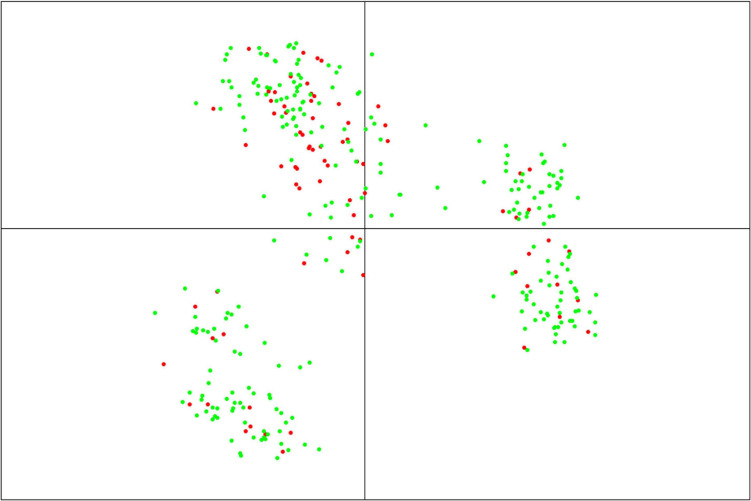
Principal component analysis (PCA) of the MAGIC population based on 19,723 SNPs. The first two axes of PCA explain 22.3 and 5.5% of the total variability, respectively. Red points represent the subset of MAGIC lines included in TP-Diverse, while green points represent the remaining MAGIC lines.

Following this “TP-Diverse” optimization, our procedure led to identify a set of 82 MAGIC lines as the smallest population subset fulfilling the aforementioned criterion, which was used as TP along with the eight founder parents. Overall, when applied to MAGIC populations, the four optimized TPs spawned similar predictive abilities across the three site-by-season combinations (Figure 2) and consequently the genetic makeup of this TP was further investigated. The genetic relationships between TP-Diverse and the remaining MAGIC lines was assessed conducting a principal component analysis (PCA) on genetic data, which pointed out that the first two principal components explain 22.3 and 5.5 percent of the total genetic variability of the MAGIC population, respectively (Figure 3). PCA shows three main clusters of MAGIC lines and corroborates that individuals included in the TP-Diverse are representative of the whole diversity of MAGIC lines (red points).

**FIGURE 4:**
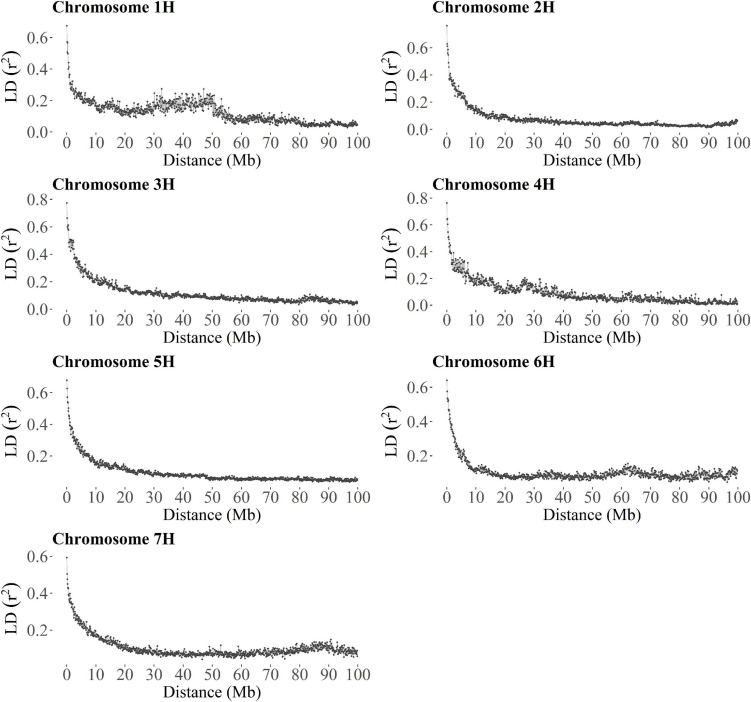
Extent of the average linkage disequilibrium in TP-Diverse. For each barley chromosome, each point shows the average **r_2_** computed in 100 kb windows as a function of marker distance.

In segregating families and collections of mostly unrelated accessions, a large number of molecular markers is often needed to capture the effects of all QTLs or alternatively, strong linkage disequilibrium (LD) between markers and causative variants that control the traits of interest is desirable to achieve high values of predictive ability in GP (Lorenzana and Bernardo, 2009; Heffner et al., 2011; Norman et al., 2018). Consequently, the extent of LD was investigated in TP-Diverse to assess its correlation with the predictive ability values of GP models. Firstly, SNP markers of the barley 50 K SNP chip used to fingerprint the whole MAGIC population were lifted over to the new barley reference sequence (Monat et al., 2019) and secondly, the average extent of *r*^2^ was computed for each barley chromosome. Overall, a large fraction of the 44,040 SNPs of the barley 50 k SNP chip were lifted over and 18,248 out 19,723 polymorphic SNPs unambiguously mapped to the reference sequence of barley (Supplementary Table 2) were used to estimate the decay of average LD computed in bins of 100 kb (Figure 4). This analysis indicated that across the seven barley chromosomes *r*^2^ decays relatively slowly as SNPs mapped more than 10 Mbp apart show *r*^2^ values of circa 0.2, while the average *r*^2^ values of markers within 1 MB or less exceed 0.4 (Figure 4). Considering the average number of markers per chromosome (Supplementary Table 2), the levels of LD measured in TP-Diverse are sufficiently high and higher marker densities might not significantly increase the predictive ability of GP models fitted in our MAGIC population of barley as empirically observed in other crops (Norman et al., 2018). Overall, the predictive ability values obtained with GP models fitted with the three optimization methods are substantially equivalent to the prediction accuracy obtained with TP-Diverse (Figure 2) and consequently this latter TP was chosen for fitting further single- and multi-environment GP models.

### Using the Optimized TP for Fitting SE-GP and ME-GP Models

Field trials of TP-Diverse were organized in nine site-by-season combinations and phenotypic data for GY and DH were collected using common phenotyping protocols, while the remaining set of MAGIC lines were used in Fio16IN, Fio17IN and Mar16IN as VP (Table 3). Alpha-lattice experimental designs were adopted for all field trials and mixed linear models were used to compute adjusted means of GY and broad sense heritability (H^2^) for each site-by-season combination considering genotypes as random variables (BLUPs) (Table 3). This analysis indicated that H^2^ varies significantly across the nine field trials and spans from 0.805 in Kon19IN to 0.122 in Mar16IN (Table 3). The adjusted means of GY were subsequently used as phenotypes for fitting GP models along with genotypic information.

**TABLE 3:** Summary of field trials carried out for phenotyping TP and VP for GY.

Acronym	Site	Country	Growing season	Populations	H^2^
Fio16IN	Fiorenzuola d’Arda	Italy	2015–2016	TP and VP	0.660
Fio17IN	Fiorenzuola d’Arda	Italy	2016–2017	TP and VP	0.472
Fio18IN	Fiorenzuola d’Arda	Italy	2017–2018	TP	0.532
Fio18LN	Fiorenzuola d’Arda–Low Nitrogen	Italy	2017–2018	TP	0.395
Fio19IN	Fiorenzuola d’Arda	Italy	2018–2019	TP	0.652
Fio19LN	Fiorenzuola d’Arda–Low Nitrogen	Italy	2018–2019	TP	0.663
Mar16IN	Marchouch	Morocco	2015–2016	TP and VP	0.122
Ada19IN	Adana	Turkey	2018–2019	TP	0.737
Kon19IN	Konya	Turkey	2018–2019	TP	0.805

*For each site-by-season combination, the estimates of broad sense heritability (H^2^) of GY were reported. H^2^ was computed for the whole panel of MAGIC lines for Fio16IN, Fio17IN and Mar16IN.*

**FIGURE 5:**
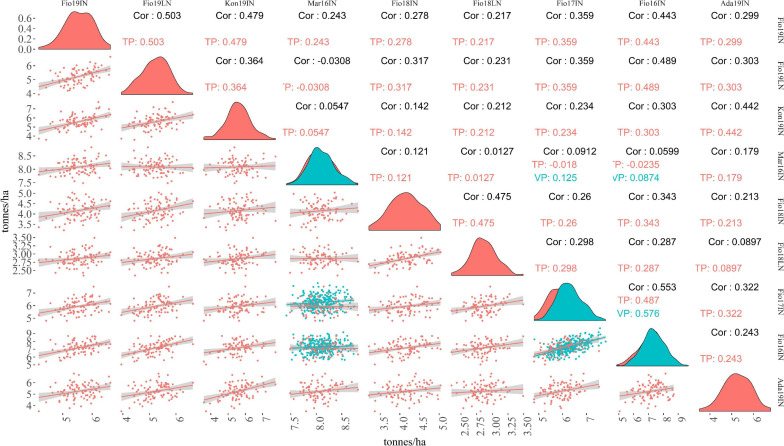
Pairwise correlations of GY obtained in the nine site-by-season combinations for TP and VP. Numbers reported in black, red, and blue on the upper graph show pairwise Pearson correlations computed between adjusted means of GY for the whole set of lines tested, TP and VP, respectively. The lower graph shows scatter plots of GY adjusted means computed in pairs of site-by-season combinations.

To assess the performance of MAGIC lines included in TP-Diverse, across different locations and years, a pairwise correlation analysis of the adjusted means of GY computed in the nine site-by-season combinations considered in this study was carried out (Figure 5). The correlations of GY across environments spanned from −0.030 to 0.553 and, as expected, values were higher between field trials carried out in the same environments but in different years, while lower values were observed among Mar16IN and other site-by-season combinations, corroborating the hypothesis that the climatic peculiarity of this environment imposes higher levels of stress to MAGIC lines (Figure 5). Similarly, the adjusted means of GY computed in Fio18LN exhibited lower correlation values with other site-by-season combinations (Figure 5). These adjusted means of GY were used to train SE-GP and ME-GP models using “TP-Diverse.” For each site-by-season combination, phenotypic and genotypic data were standardized, and nine different SE-GP models were fitted using GB and GK statistical models (Table 4). As expected after standardization, for models fitted using GB, the summation of variance components was circa 1 (Table 4), while the distribution of the residuals after fitting all GP models to the nine site-by-season combinations was approximately normal. The analysis of variance components of SE-GP models showed that the values of error variance in GK models are lower than those obtained for the corresponding GB models (Table 4), and similarly in GK models the values of genetic component variance are always higher than the corresponding quantities computed for GB models (Table 4).

**TABLE 4:** Variance components of SE-GP models fitted using GBLUP (GB) and GK statistical model.

Site-by-season combination	GB		GK		
	Genetic effect variance	Residual variance	Genetic effect variance	Residual variance	
Kon19IN	0.586 (0.010)	0.557 (0.045)	0.660 (0.016)		0.489 (0.068)
Mar16IN	0.467 (0.089)	0.719 (0.067)	0.590 (0.013)		0.588 (0.078)
Fio18IN	0.491 (0.059)	0.560 (0.029)	0.632 (0.086)		0.455 (0.043)
Fio18LN	0.412 (0.048)	0.752 (0.050)	0.544 (0.000)		0.611 (0.069)
Fio17IN	0.537 (0.072)	0.480 (0.016)	0.655 (0.084)		0.417 (0.041)
Fio16IN	0.618 (0.066)	0.336 (0.094)	0.680 (0.065)		0.348 (0.011)
Ada19IN	0.561 (0.086)	0.543 (0.036)	0.654 (0.019)		0.498 (0.070)
Fio19IN	0.480 (0.079)	0.651 (0.049)	0.659 (0.005)		0.469 (0.054)
Fio19LN	0.479 (0.058)	0.566 (0.024)	0.632 (0.083)		0.446 (0.041)

*For each site-by-season combination, the estimated variance components of genetic effects and residuals fitted with GB and GK models are reported, while bracketed numbers point out the corresponding standard deviation.*

The adjusted means of GY computed at the nine site-by-season combinations were used to fit ME-GP, particularly three models were fitted, which were named “Multi-environment, main genotypic effect” (MM), “Multi-environment, single variance GxE deviation” (MDs) (Jarquín et al., 2014) and “Multi-environment, environment specific variance GxE deviation” (MDe) (López-Cruz et al., 2015) following recent model nomenclature (Bandeira e Sousa et al., 2017). Similarly to SE-GP models, MM, MDs, and MDe models were fitted using GB and GK methods and totally six model method combinations were used to fit multi-environment predictions. The analysis of variance components showed that for all three models (MM, MDs, and MDe), GK methods exhibit lower values of the estimated residual variances pointing out a better model fitting (Table 5). Moreover, model comparisons showed that the inclusion of the interaction term (GxE) in MDe model induces a reduction in the estimated residual variance for GY compared to MM models either using GB or GK methods, but MDs models fitted better the data compared to MDe. For the MDe models, the residual variance components of MDe-GK were smaller than those of the MDe-GB, whereas the estimated variance components for the genetic main effect and genetic environment specific effect variances were higher for the GK than for the GB (Table 5).

**TABLE 5:** Variance components of ME-GP models fitted using GBLUP (GB) and RKHS along with the Gaussian Kernel (GK) methods.

Component	Environment	GB	GK
**Multi-environment, main genotypic effect (MM) model**			
Residual ($\sigma_{e}^{2}$)	–	0.758 (0.047)	0.746 (0.045)
Genetic main effect ($\sigma_{\mu 0}^{2}$)	–	0.249 (0.069)	0.373 (0.088)
**Multi-environment, single variance GxE deviation (MDs) model**			
Residual ($\sigma_{e}^{2}$)	–	0.516 (0.056)	0.389 (0.071)
Genetic main effect ($\sigma_{\mathrm{u0}}^{2}$)	–	0.281 (0.077)	0.374 (0.089)
Genetic interaction effect ($\sigma_{ue}^{2}$)	–	0.247 (0.066)	0.589 (0.140)
**Multi-environment, environment specific variance GxE deviation**			
**(MDe) model**			
Residual ($\sigma_{e}^{2}$)	–	0.602 (0.016)	0.592 (0.018)
Genetic main effect ($\sigma_{\mathrm{u0}}^{2}$)	–	0.292 (0.026)	0.402 (0.031)
Genetic environment specific effect ($\sigma_{uEj}^{2}$)	Ada19IN	0.251 (0.054)	0.353 (0.083)
	Fio16IN	0.035 (0.027)	0.054 (0.046)
	Fio17IN	0.010 (0.066)	0.024 (0.023)
	Fio18LN	0.062 (0.054)	0.116 (0.085)
	Fio18IN	0.007 (0.006)	0.018 (0.015)
	Mar16IN	0.549 (0.085)	0.873 (0.122)
	Kon19IN	0.217 (0.050)	0.312 (0.079)
	Fio19LN	0.008 (0.007)	0.053 (0.018)
	Fio19IN	0.004 (0.003)	0.055 (0.011)

*For each of the three regression models (MM, MDs and MDe), the estimated variance components fitted with GB and GK methods are reported, while bracketed numbers point out the corresponding standard deviation of variance component estimates.*

### Predictive Ability of ME-GP Models With GB and GK Methods

**FIGURE 6:**
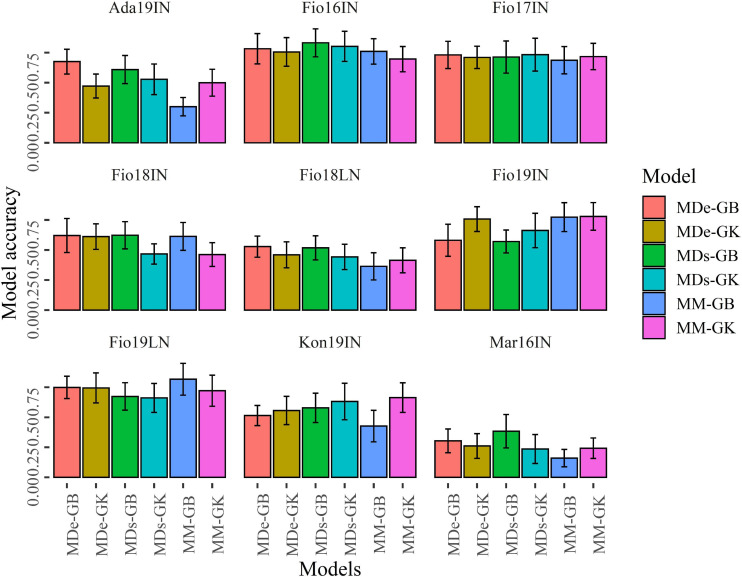
Bar plots of the predictive ability values obtained with CV2. Bar plots show the mean correlation between observed and predicted values of GY obtained with 100 random CV2 partitions for MM, MDs and MDe models implemented with GBLUP (GB) and Gaussian Kernel (GK) methods. Error bars point out the standard deviation of predictive ability values.

The predictive ability of MM, MDs, and MDe models implemented using GB and GK methods was estimated with cross-validation 1 (CV1) and cross-validation 2 (CV2) schemes using 100 random partitions. For each of the six multi-environment model-method combinations, the values of predictive ability for CV1 and CV2 schemes were obtained for the set of 100 random partitions, which were used to compute the average predictive ability and the associated standard deviation. Overall, CV2 showed that in four site-by-season combinations (Fio16IN, Fio17IN, Fio19IN, and Fio19LN) the predictive ability is generally higher and exceed 0.70 for certain ME-GP models, while for Mar16IN the six model-method combinations exhibit, on average, the lowest values of predictive ability as for this site-by-season combination the lowest values of 0.161 and 0.236 were observed for MM-GB and MDs-GK models, respectively (Figure 6 and Supplementary Table 3).

**FIGURE 7:**
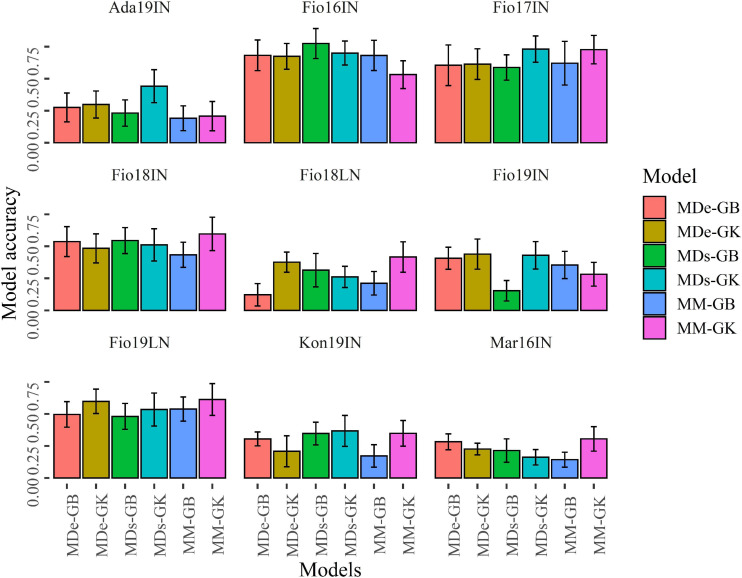
Bar plots of the predictive ability values obtained with CV1. Bar plots show the mean correlation between observed and predicted values of GY obtained with 100 random CV1 partitions for MM, MDs, and MDe models implemented with GBLUP (GB) and Gaussian Kernel (GK) methods. Error bars point out the standard deviation of predictive ability values.

As in most of the case, the standard deviations associated to the values of predictive ability were overlapping (Figures 6, 7), Welch’s *t*-tests were applied to determine whether pairwise comparisons of predictive ability values obtained with ME-GP models were statistically different (Supplementary Figures 1, 2). CV2 experiments showed that in Fio17IN the values of predictive ability computed with the six-model method combinations were comparable except for MM-GB, which was significantly lower than the predictive ability of MDs-GK, while in Fio16IN the predictive ability of MM-GK was significantly lower than the predictive ability obtained with the remaining model-method combinations (Figure 6 and Supplementary Figure 2). In Fio16IN, CV2 showed that MDe-GB and MDe-GK have similar performance and significantly higher values of predictive ability compared to MM models, either implemented with GB or GK statistical methods (Figure 6, Supplementary Table 3, and Supplementary Figure 2). In Ada19IN the best model predictive ability using CV2 scheme was obtained with MDe-GB, while for Fio18LN the best values of predictive ability were obtained with MDe-GB and MDs-GB models. Overall, CV2 experiments indicated that in four out nine site-by-season combinations (Fio16IN, Fio17IN, Fio18IN, and Mar16IN) MDe-GB and MDe-GK models have higher values of predictive ability compared to MM models, either implemented with GB or GK statistical methods (Figure 6, Supplementary Table 3, and Supplementary Figure 2). Differently, Fio19IN, Fio19LN, and Kon19IN deviate from this trend as for these site-by-season combinations the values of predictive ability for MM models were higher (Supplementary Table 3). In Fio19IN, MM-GB and MM-GK had the higher predictive ability values along with MDe-GK, while for Fio19LN the higher value of predictive ability was found for MM-GB.

The values of predictive ability obtained for random CV1 decreased (Figure 7 and Supplementary Table 4) as compared with those computed for CV2 for all models. Similarly to the results obtained for CV2, CV1 experiments indicated that in four site-by-season combinations (Fio16IN, Fio17IN, Fio18IN, and Fio19LN) the predictive ability of GP-ME models is generally higher than the values of predictive ability observed in other site-by-season combinations for all models. MDs-GB and MD-GK yielded the higher values of predictive ability in Ada19IN, Fio16IN, and Fio17IN, respectively. In Fio18IN, Fio18LN, Mar16IN, and Fio19LN, the higher predictive ability values were found for MM-GK, although in this latter site-by-season combination the accuracy of MDe-GK does not differ significantly (Supplementary Figure 1). In Fio19IN, the highest values of predictive ability were obtained for MDe-GB and MD-GK models (Figure 7 and Supplementary Figure 1).

## Discussion

### Broadening the Use of MAGIC Populations for Plant Breeding

Multi-parent Advanced Generation Intercrosses populations were conceived to improve precision and efficiency of QTL mapping in plants and animals as they allow overcoming limitations of biparental populations and association mapping panels (Huang et al., 2015). In cereal crops, these experimental populations have been extensively used for research purpose and contributed to dissecting the genetic bases of several traits among which biotic stress resistance (Stadlmeier et al., 2018; Jiménez-Galindo et al., 2019; Riaz et al., 2020), GY, grain quality (Zaw et al., 2019) and DH (Afsharyan et al., 2020). Recently, these genomic resources have been established in barley to investigate the effects of epistasis and environmental interactions on flowering time (Mathew et al., 2018; Afsharyan et al., 2020), further broadening the original scope for which they were devised.

In the present study, we constructed a new MAGIC population shuffling alleles of winter 6-rowed barley varieties, and demonstrated that, along with biparental populations and collections of mostly unrelated accessions, these genomic resources might be used to train GP models with high predictive ability and might speed up barley breeding. Under this point of view, the large number of MAGIC populations developed in the last years in several crops (Kover et al., 2009; Rebetzke et al., 2014; Mathew et al., 2018; Stadlmeier et al., 2018) can be considered as untapped resources that would contribute to further strengthening and stimulating the application of GP in plant breeding. On the other side, *de novo* creation of MAGIC populations to train GP models for actual breeding purposes is hampered because of their time consuming and costly development, which requires to intermate and self-fertilize the founder parents for several cycles. The results presented in this study show that these limitations might be softened using doubled haploid technology, which allows to short self-fertilization stages to obtain fully homozygous lines. Similarly, speed breeding might contribute to accelerating the development of new MAGIC populations (Watson et al., 2018).

To examine the genetic relationship between the whole set of MAGIC and the subset of lines included in the “TP-Diverse,” a PCA was carried out using 19,723 SNPs, which detected genetic structure in the MAGIC population and three main clusters of individuals. The nature of these clusters is unclear, but it is plausible that they might reflect subgroups of individuals showing segregation distortion for one or more founders. In our eight-way MAGIC population, the expected segregation rate of the eight founder haplotypes is 1:1:1:1:1:1:1:1, but the haplotypes of some founders (e.g., Dea) deviate from the expected ratio (**Data not shown**). Segregation distortion is a common phenomenon that occurs in MAGIC populations as pointed out in other studies (Sannemann et al., 2018). Although this did not hamper our ability to train GP models with this population, this phenomenon might explain the genetic structure pointed out with PCA.

Overall, the use of SE-GP and ME-GP models trained with MAGIC populations might find effective applications when the diversity of BPs originates from the same parents included in the founder set. In this case, GP models based on MAGIC populations might be applied to select the best offspring from crosses obtained with the MAGIC founders.

### Benchmarking of Different TPs to Improve the Predictive Ability of GP Models

The composition of TPs and their genetic relationship with BPs affect the predictive ability of GP models as pointed out in several studies (Desta and Ortiz, 2014; Norman et al., 2018; Edwards et al., 2019) and to date several algorithms for optimizing TPs have been developed to increase the predictive ability of GP models (Akdemir et al., 2015). Untargeted and targeted optimization criteria based on GBLUP have been so far developed and tested in biparental populations and panel of mostly unrelated accessions. Nevertheless, the use of these optimization methods in actual breeding programs is hampered as the optimization process can lead to different optimized TP per each trait of interest. These optimization algorithms require *a priori* information (knowledge of the BP genotypes and traits for which GP models must be developed) and output trait-dependent TPs (Akdemir et al., 2015). Moreover, in real breeding programs, BPs change over time and it might be difficult to implement these optimization procedures. Previous studies have shown that the relatedness between TPs and BPs has a large impact on the predictive ability of GP models, which can be improved increasing the genetic diversity of TPs (Norman et al., 2018). In fact, when the TP exhibits a narrow genetic diversity, low values of the predictive ability are often obtained in GP as it becomes impossible to predict all the marker effects that contribute to determining the phenotypic variations (Norman et al., 2018). Following these empirical findings, in this study we assembled a TP of 90 barley genotypes, which was named “TP-Diverse,” maximizing the genetic diversity among MAGIC lines and assessing its predictive ability using random CV schemes. Surprisingly, the predictive ability obtained with TP-Diverse was comparable with the predictive ability of GP models trained with the other three optimized TPs used in this study (Figure 2). One of the main advantages of using this approach is that the criterion adopted to assemble “TP-Diverse” depends only on genetic data and does not generate trait-dependent TPs. On the other side, in this study we have not developed mathematical models to demonstrate or justify the rationale of this empirical criterion and consequently its validity should be further validated in other studies.

### Fitting SE-GP and ME-GP Models Using the MAGIC Population of Barley

Several empirical analyses have been conducted to benchmark the predictive ability of different GP models in barley, maize and wheat panels of mostly unrelated accessions, biparental populations of *A. thaliana* and diallel crosses of maize and wheat to predict GY and other traits (Heslot et al., 2012). In this study, we presented another empirical analysis to assess the most promising GP models for MAGIC populations, implementing CV schemes for estimating the standard deviation of predictive ability values.

Three out five models fitted in this study (BayesA, BayesB, and BL) belong to the group of so called “Bayesian alphabet,” which denotes Bayesian linear regressions that differ in their prior density distribution (Gianola, 2013). In these Bayesian regression models, the prior density distribution assigned to marker effects controls the shrinkage of estimates and then different priors induce different types of shrinkage of marker effects. In the original description both BayesA and BayesB were introduced as hierarchical structures (Meuwissen et al., 2001) and it was later demonstrated that BayesA adopts a scaled t-distribution prior, while BayesB adopts priors that are mixtures of a peak in the vicinity of zero and of a continuous density priors (e.g., t, or normal density distribution) (Gianola et al., 2009). BL adopts a double exponential prior density distribution, which behaves similar to that of BayesA as both priors used in these models do not allow marker effects to be equal to zero and shrink estimates of the remaining marker effects. While the priors adopted in BL and BayesA prevent to have marker effects equal to zero, the prior used in BayesB allows to have null marker effects. The rationale of this prior is that in GP many markers might have a null contribution to the observed phenotypic variation. Although marker effects might be estimated differently, the predictive ability of the Bayesian models fitted in this study does not differ significantly (Figure 1). Moreover, our empirical analysis shows that the predictive ability of Bayesian models fitted to MAGIC populations is comparable with that of GB and GK models (Figure 1). Several empirical analyses have been carried out in cereal crops to highlight advantages and limits of different whole genome regression methods. In rice, SE-GP models fitted with BayesA, GB, and GK for three traits were compared using a reference panel of 284 accessions under different linkage disequilibrium scenarios (Ben Hassen et al., 2018). These results showed that under high linkage disequilibrium scenarios GK models slightly outperform GB in terms of prediction ability. Differently, when a subset of rice reference panel was used to predict the performance of 97 advanced lined derived from biparental crosses, GK and GB prediction ability showed comparable results for the three traits considered (Ben Hassen et al., 2018). Anyway, the results obtained in this study are limited to one (complex) trait and it might plausible that for simpler traits GP models fitted in MAGIC might have different trend of the predictive ability.

Beyond SE-GP models, in this study we used the MAGIC population of barley to fit three different ME-GP models, two of which (MDs and MDe models) include terms for incorporating GxE interaction. In plant breeding, multi-environment field trials are routinely carried out to evaluate and exploit GxE interaction as it contributes to creating high-yielding genotypes. Consequently, modeling GxE interaction in GP has the potential to differentiate marker effects. MDe models used in this study (López-Cruz et al., 2015; Bandeira e Sousa et al., 2017) partition marker effects in main effects, that is effects that are stable across environments and environment-specific effects, that is interaction effects between markers and specific genotypes. As pointed out in other studies, MDe models are known to be more efficient when used along with sets of environments that have positive correlations. This limit arises as the pairwise correlation between environments is represented by the variance of the main marker effects, which in turn forces the co-variance between a pair of environments to be positive (López-Cruz et al., 2015; Bandeira e Sousa et al., 2017). This requirement is not trivial and might not allow to fit correctly MDe models. In our study, the adjusted means of GY in Mar16IN showed low or negative correlation with the other site-by-season combinations tested in this study and this might be the reason for which we have found that MDs models fit better the data, particularly when used in combination with the non-linear GK.

GP models based on reproducing kernel Hilbert Space along with the non-linear GK have the potential to capture non-additive genetic effects and potentially might outperform GB in terms of model fitting and predictive ability. In maize and wheat, comparison between the same GP models fitted with GB and the nonlinear GK for GY, unveiled that the latter method outperforms GB in terms of predictive ability in both single environment and multi-environment models (Cuevas et al., 2016; Bandeira e Sousa et al., 2017). In cereal crops, GY is a complex trait controlled by nonlinearity effects between genotypes and phenotypes owing to epistasis, environmental interactions (Bandeira e Sousa et al., 2017; Cuevas et al., 2018) and other interactions that are not considered in standard quantitative genetic models (Gianola et al., 2006). GK models have the potential to capture small and complex interactions, which are more evident in quantitative traits and this can explain the higher prediction ability of GK for GY. The empirical analysis presented in this study using barley MAGIC population corroborates that, for complex traits like GY, the predictive ability of GK outperforms that of GB. Overall, considering the number of models and methods fitted and the extensive field trials carried out across the Mediterranean, this study has delivered the most comprehensive empirical analysis of GP models fitted with MAGIC populations.

## Data Availability Statement

The datasets presented in this study can be found in online repositories. The names of the repository/repositories and accession number(s) can be found in the article/Supplementary Material.

## Author Contributions

AF designed and supervised the research along with the help of EI and LC. AF wrote the manuscript along with DP and significant contributions from AV, EI, HO, AC, AT, GV, LC, and AP. DP performed the research, while GV, AT, and SD developed the MAGIC population. AV, HO, EI, AC, SD, AT, İK, AP, and DP carried out field trials and plant phenotyping. All authors contributed to the article and approved the submitted version.

## Conflict of Interest

The authors declare that the research was conducted in the absence of any commercial or financial relationships that could be construed as a potential conflict of interest.

## Abbreviations

DH: Days-to Heading
PH: Plant Height
GY: Grain Yield
MAGIC: Multi-parent Advanced Generation Inter-crosses
TP: Training Population
BP: Breeding Population
GP: Genomic Prediction
GBLUP: Genomic Best Linear Unbiased Prediction
RKHS: Reproducing Kernel Hilbert Space
GK: Gaussian Kernel
LD: Linkage Disequilibrium
PCA: Principal Component Analysis
QTL: Quantitative Trait Locus
GEBV: Genomic Estimated Breeding Value
SNP: Single Nucleotide Polymorphism
SE-GP: Single Environment Genomic Prediction
ME-GP: Multi Environment Genomic Prediction.
