# Supplementary material for: Genomic Prediction of Grain Yield in a Barley MAGIC Population Modeling Genotype per Environment Interaction
Source: Front Plant Sci. 2021 May 24;12:664148. doi: 10.3389/fpls.2021.664148 (PMC8183822; doi:10.3389/fpls.2021.664148)
Supplement: Supplementary file 1 [file Data_Sheet_1.docx]

Supplementary Material

**Supplementary Figure 1.** Heatmap of *P*-values obtained applying Welch’s *t*-test to accuracy parameters (means and standard deviations) obtained from pairs of ME-GP models using CV1 schemes. Red tiles in the heatmap point out pairs of models that exhibit significantly different accuracy (*P*-values<0.05), while blue tiles point out pairs of models that do not show different model accuracy.


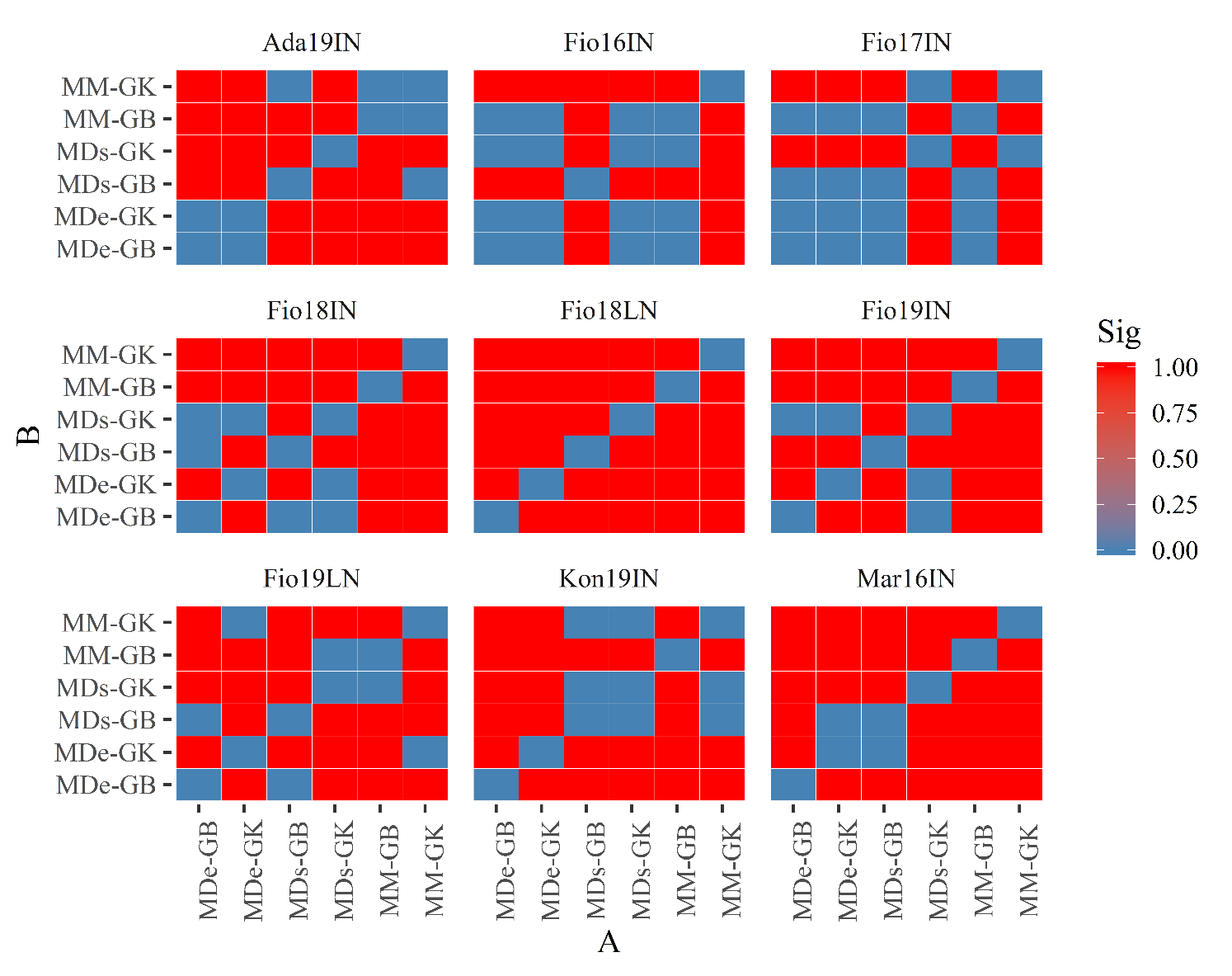


**Supplementary Figure 2.** Heatmap of *P*-values obtained applying Welch’s *t*-test between model accuracy values obtained using CV2 schemes for each ME-GP model. Red tiles in the heatmap point out pairs of models that exhibit significantly different model accuracy values (*P*-values<0.05), while blue tiles point out pairs of models that do not show different model accuracy.


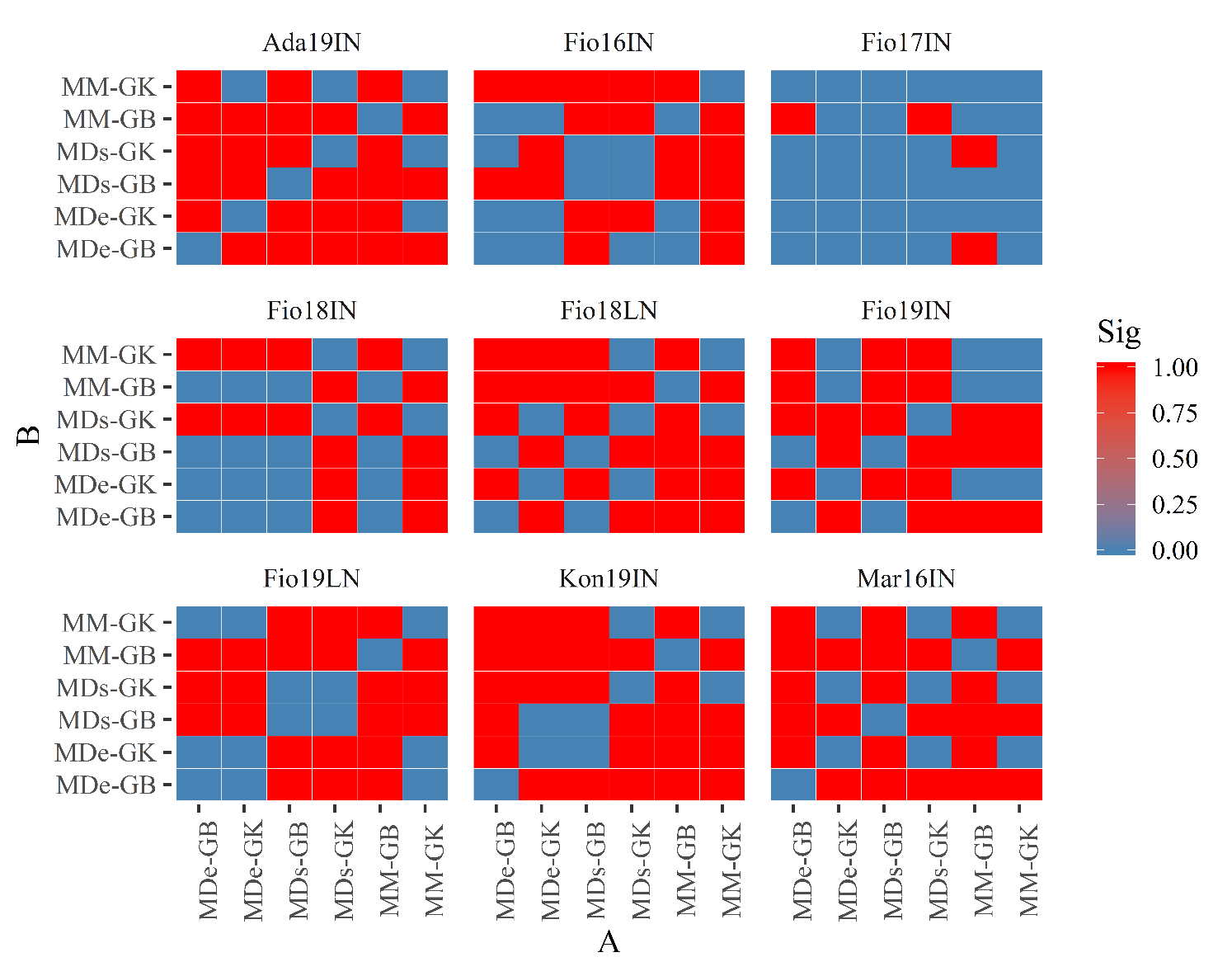


**Supplementary Table 1: CV of different SE-GP models fitted using MAGIC population.** Predictive ability values and associated standard deviation of SE-GP models (reported within brackets), computed in Fio16IN, Fio17IN and Mar16IN using genomic best linear unbiased predictor (GB), RKHS along with the Gaussian Kernel (GK), BayesA, BayesB and Bayesian Lasso (BL) models as a function of TP sizes.

| Environment | TP size | BayesA | BayesB | BL | GB | GK |
| --- | --- | --- | --- | --- | --- | --- |
| Fio16IN | 80 | 0.511 (0.037) | 0.51 (0.039) | 0.516 (0.038) | 0.512 (0.035) | 0.501 (0.038) |
| Fio16IN | 90 | 0.52 (0.033) | 0.519 (0.039) | 0.522 (0.033) | 0.519 (0.033) | 0.517 (0.037) |
| Fio16IN | 100 | 0.527 (0.029) | 0.528 (0.038) | 0.528 (0.035) | 0.526 (0.033) | 0.517 (0.033) |
| Fio16IN | 110 | 0.534 (0.031) | 0.533 (0.039) | 0.539 (0.035) | 0.533 (0.034) | 0.526 (0.035) |
| Fio16IN | 120 | 0.547 (0.035) | 0.54 (0.036) | 0.54 (0.032) | 0.539 (0.037) | 0.531 (0.036) |
| Fio16IN | 130 | 0.546 (0.039) | 0.543 (0.037) | 0.549 (0.031) | 0.546 (0.035) | 0.541 (0.036) |
| Fio16IN | 140 | 0.553 (0.035) | 0.55 (0.037) | 0.553 (0.037) | 0.55 (0.038) | 0.548 (0.038) |
| Fio16IN | 150 | 0.56 (0.034) | 0.558 (0.035) | 0.558 (0.037) | 0.557 (0.037) | 0.547 (0.036) |
| Fio16IN | 160 | 0.557 (0.031) | 0.559 (0.042) | 0.558 (0.035) | 0.564 (0.036) | 0.547 (0.038) |
| Fio17IN | 80 | 0.509 (0.036) | 0.506 (0.033) | 0.514 (0.032) | 0.514 (0.032) | 0.504 (0.04) |
| Fio17IN | 90 | 0.525 (0.032) | 0.526 (0.03) | 0.523 (0.034) | 0.516 (0.033) | 0.511 (0.033) |
| Fio17IN | 100 | 0.53 (0.035) | 0.528 (0.032) | 0.532 (0.034) | 0.526 (0.032) | 0.526 (0.035) |
| Fio17IN | 110 | 0.53 (0.037) | 0.534 (0.033) | 0.537 (0.027) | 0.529 (0.036) | 0.532 (0.032) |
| Fio17IN | 120 | 0.543 (0.033) | 0.544 (0.032) | 0.544 (0.028) | 0.545 (0.034) | 0.533 (0.037) |
| Fio17IN | 130 | 0.548 (0.035) | 0.542 (0.039) | 0.551 (0.035) | 0.545 (0.035) | 0.54 (0.032) |
| Fio17IN | 140 | 0.553 (0.04) | 0.553 (0.036) | 0.546 (0.037) | 0.553 (0.036) | 0.546 (0.034) |
| Fio17IN | 150 | 0.561 (0.032) | 0.555 (0.035) | 0.557 (0.034) | 0.562 (0.035) | 0.554 (0.04) |
| Fio17IN | 160 | 0.562 (0.039) | 0.558 (0.039) | 0.566 (0.039) | 0.56 (0.037) | 0.559 (0.04) |
| Mar16IN | 80 | 0.16 (0.052) | 0.165 (0.055) | 0.151 (0.062) | 0.166 (0.059) | 0.151 (0.06) |
| Mar16IN | 90 | 0.169 (0.054) | 0.17 (0.054) | 0.176 (0.049) | 0.169 (0.055) | 0.171 (0.056) |
| Mar16IN | 100 | 0.186 (0.041) | 0.183 (0.05) | 0.181 (0.055) | 0.174 (0.055) | 0.182 (0.044) |
| Mar16IN | 110 | 0.199 (0.052) | 0.185 (0.05) | 0.177 (0.056) | 0.179 (0.059) | 0.189 (0.05) |
| Mar16IN | 120 | 0.194 (0.049) | 0.191 (0.045) | 0.190 (0.057) | 0.193 (0.054) | 0.191 (0.054) |
| Mar16IN | 130 | 0.203 (0.054) | 0.206 (0.051) | 0.190 (0.058) | 0.2 (0.049) | 0.198 (0.051) |
| Mar16IN | 140 | 0.209 (0.049) | 0.205 (0.048) | 0.209 (0.051) | 0.201 (0.049) | 0.194 (0.053) |
| Mar16IN | 150 | 0.211 (0.056) | 0.208 (0.055) | 0.211 (0.05) | 0.206 (0.056) | 0.204 (0.048) |
| Mar16IN | 160 | 0.22 (0.049) | 0.213 (0.054) | 0.211 (0.051) | 0.217 (0.052) | 0.205 (0.041) |

Supplementary Table 2: Distribution of polymorphic SNPs mapped in the seven barley chromosomes. 18,248 out 19,723 polymorphic SNPs were unambiguously mapped to the barley reference sequence and used to compute r^2^ to estimate the extent of LD.

| Chromosome | Number of SNPs | Average SNPs per Mb |
| --- | --- | --- |
| Chromosome 1H | 2095 | 4.00 |
| Chromosome 2H | 2281 | 3.38 |
| Chromosome 3H | 2855 | 4.54 |
| Chromosome 4H | 1444 | 2.31 |
| Chromosome 5H | 3589 | 5.99 |
| Chromosome 6H | 2615 | 4.56 |
| Chromosome 7H | 3369 | 5.31 |
| Total | 18248 | 4.36 |

Supplementary Table 3: Mean correlation between observed and predicted values of GY (average of 100 random CV partitions) computed using CV2 scheme for MM, MDs and MDe models implemented with GBLUP (GB) and Gaussian Kernel (GK) methods. Numbers between brackets point out the standard deviation of model accuracy values.

| Model | MDe-GB | MDe-GK | MDs-GB | MDs-GK | MM-GB | MM-GK |
| --- | --- | --- | --- | --- | --- | --- |
| Ada19IN | 0.676 (0.103) | 0.472 (0.1) | 0.609 (0.117) | 0.528 (0.128) | 0.301 (0.076) | 0.500 (0.112) |
| Fio16IN | 0.783 (0.125) | 0.756 (0.119) | 0.832 (0.116) | 0.802 (0.125) | 0.760 (0.106) | 0.696 (0.105) |
| Fio17IN | 0.731 (0.114) | 0.711 (0.093) | 0.714 (0.134) | 0.734 (0.137) | 0.687 (0.113) | 0.718 (0.109) |
| Fio18IN | 0.621 (0.142) | 0.612 (0.106) | 0.623 (0.113) | 0.467 (0.084) | 0.613 (0.116) | 0.462 (0.099) |
| Fio18LN | 0.528 (0.088) | 0.46 (0.108) | 0.518 (0.1) | 0.443 (0.105) | 0.364 (0.113) | 0.414 (0.104) |
| Fio19IN | 0.581 (0.133) | 0.757 (0.103) | 0.571 (0.095) | 0.662 (0.143) | 0.774 (0.12) | 0.779 (0.115) |
| Fio19LN | 0.749 (0.093) | 0.744 (0.125) | 0.674 (0.114) | 0.661 (0.121) | 0.817 (0.132) | 0.721 (0.129) |
| Kon19IN | 0.515 (0.083) | 0.557 (0.118) | 0.579 (0.122) | 0.632 (0.152) | 0.427 (0.131) | 0.664 (0.123) |
| Mar16IN | 0.304 (0.098) | 0.261 (0.103) | 0.384 (0.139) | 0.236 (0.12) | 0.161 (0.072) | 0.243 (0.085) |

Supplementary Table 4: Mean correlation between observed and predicted values of GY (average of 100 random CV partitions) computed using CV1 scheme for MM, MDs and MDe models implemented with GBLUP (GB) and Gaussian Kernel (GK) methods. Numbers between brackets point out the standard deviation of predictive ability values.

| Model | MDe-GB | MDe-GK | MDs-GB | MDs-GK | MM-GB | MM-GK |
| --- | --- | --- | --- | --- | --- | --- |
| Ada19IN | 0.276 (0.113) | 0.298 (0.106) | 0.232 (0.102) | 0.442 (0.129) | 0.192 (0.097) | 0.208 (0.114) |
| Fio16IN | 0.683 (0.120) | 0.675 (0.101) | 0.775 (0.117) | 0.701 (0.094) | 0.682 (0.118) | 0.532 (0.109) |
| Fio17IN | 0.605 (0.158) | 0.614 (0.119) | 0.588 (0.099) | 0.731 (0.102) | 0.621 (0.17) | 0.727 (0.111) |
| Fio18IN | 0.537 (0.117) | 0.484 (0.113) | 0.545 (0.102) | 0.512 (0.126) | 0.434 (0.098) | 0.597 (0.13) |
| Fio18LN | 0.122 (0.088) | 0.377 (0.079) | 0.314 (0.13) | 0.261 (0.083) | 0.211 (0.092) | 0.417 (0.119) |
| Fio19IN | 0.407 (0.087) | 0.439 (0.118) | 0.153 (0.080) | 0.43 (0.107) | 0.355 (0.106) | 0.282 (0.093) |
| Fio19LN | 0.496 (0.1) | 0.599 (0.096) | 0.481 (0.101) | 0.535 (0.129) | 0.539 (0.095) | 0.613 (0.124) |
| Kon19IN | 0.305 (0.054) | 0.208 (0.121) | 0.348 (0.089) | 0.367 (0.121) | 0.173 (0.087) | 0.349 (0.1) |
| Mar16IN | 0.283 (0.062) | 0.226 (0.046) | 0.215 (0.091) | 0.162 (0.06) | 0.143 (0.059) | 0.306 (0.096) |
